# Interleukin 8 (IL-8) - a universal biomarker?

**DOI:** 10.1186/1755-7682-3-11

**Published:** 2010-06-15

**Authors:** Aamir Shahzad, Martin Knapp, Irene Lang, Gottfried Köhler

**Affiliations:** 1 Max F. Perutz Laboratories, Department of Structural Biology and Biomolecular Chemistry, University of Vienna, Vienna, Austria; 2 OnkoTec GmbH. Waidhofen/Thaya, Vienna, Austria; 3 Division of Cardiology, Department of Internal Medicine II, Vienna General Hospital, Medical University of Vienna, Vienna, Austria

## Abstract

Many clinical conditions including various types of cancers are complex and generally require invasive, laborious, expensive and time-consuming investigations for their diagnosis, treatment and follow-up. There is therefore a general need for exploring non-invasive markers in clinical medicine. Interleukin 8 (IL-8) is currently being applied in various subspecialties of medicine either for the purpose of rapid diagnosis or as a predictor of prognosis. Nevertheless, there is need for large-scale studies to substantiate accuracy and outcome. This article will summarize current evidence suggesting that Interleukin 8 (IL-8) may serve as a useful biomarker.

## Background

Cancer is the second leading common cause of death. Nearly, all types of cancer present diagnostic challenges [1].Such diagnostic difficulty can delay treatment resulting in excess mortality and highlight the need for better biomarker. In addition, infectious diseases are major health care problem in developing countries. While there are diagnostic tests available for infectious diseases, these tests are laborious and time consuming [2].Due to delay in diagnosis, physicians commonly start early broad-spectrum antibiotics which may result in development of multiple drug resistant strains of microorganisms. Thus new biomarkers for disease must have a high sensitivity and specificity.

Cytokines are important mediators of inflammation and are associated with pathogenesis of many inflammatory diseases. Cytokines are in clinical use as non-invasive diagnostic markers, for disease prognosis and to monitor treatment response. Interleukin 8 (IL-8) is a member of the CXC chemokine subfamily [3] and is produced by blood cells and many types of tissues [4]. Neutrophil are major specific target for IL-8 action. Many pathophysiological actions of IL-8 depend on activation of neutrophils [4]. IL-8 is in routine use as a marker for various clinical conditions (Table 1). In this article, we will summarize some promising uses of Interleukin 8 (IL-8) as a marker for diseases.

**Table 1:** Promising use of interleukin 8 (IL-8) in various fields of medicine

Field	proposed Use^a^	Reference
Urology and Nephrology	Marker for chronic prostatitis	[6]
	Marker for acute pyelonephritis	[8]
		
Hematology	Marker for non-Hodgkin's lymphoma	[17]
		
Oncology	Marker for urinary bladder cancer	[5]
	Therapeutic target to control cancer growth and metastasis	[18]
		
Pediatrics	Marker for nasocomial bacterial infections in neonatal intensive care unit (NICU)	[15,16]
	Marker for vesicoureteral reflux (VUR).	[9]
		
Microbiology	Detection of pulmonary infections	[11]
	Detection of osteomyelitis	[12]
		
Nuclear Medicine	^99m^Tc-labeled IL-8 scintigraphy for diagnosis of clinical conditions	[10]

a, Further large scale studies are required.

### Marker for Urinary bladder cancer

Higher Interleukin 8 (IL-8) levels are present in patients with invasive bladder cancer patients when compared to individuals with superficial bladder cancer with 71% assay sensitivity and specificity. In addition, urinary IL-8 levels can discriminate between superficial bladder cancer (SBC) and muscle invasive bladder cancer (MIBC) [5]; however, low assay sensitivity (62%) suggests urinary IL-8 is not a good marker for early diagnosis of bladder cancer, but may serve as a predictor of prognosis [5].

### Marker for Prostatitis

Increased IL-8 levels were detected in expressed prostatic secretions (EPS) of chronic prostatitis (CP) [6]. In addition, higher IL-8 levels were detected in prostatic secretions, ejaculate and serum from patients with latent CP [7]. Thus increased expression of cytokines in EPS may serve as a valuable diagnostic marker for CP. Other cytokines such as IL-2 and IL-10 were also detected in EPS [6]. A panel of assay including cytokines detection in EPS can serve as good marker for CP [6].

### Marker for acute Pyelonephritis

Serum and urine IL-6 and IL-8 levels may be useful for rapid detection of acute pyelonephritis in febrile children [8]. This appears promising especially in pediatric cases of urinary tract infections (UTI) which often present with insufficient clinical manifestations thus making rapid diagnosis of acute pyelonephritis extremely challenging. Through rapid diagnosis and early treatment of acute pyelonephritis, serious complications such as renal scarring leading to hypertension and renal insufficiency can be avoided. A positive correlation was shown between serum and urine levels of IL-6, IL-8, and C-reactive protein (CRP), WBC and fever in children with acute pyelonephritis [8].IL-8 levels in serum has 81% sensitivity and 78% specificity for the diagnosis of acute pyelonephritis, while, urine IL-8 levels showed 83% sensitivity and 83% specificity [8].

### Marker for Vesicoureteral reflux (VUR)

Vesicoureteral reflux (VUR) is a condition which predisposes children to recurrent UTI which may lead to hypertension and renal insufficiency [9]. Currently, VUR is diagnosed using expensive and invasive methods which carry the risk of radiation exposure. Though, ultrasound is non invasive technique but it has low sensitivity for diagnosis of VUR [10,11]. Emmanouil et al found a concentration at 5pg/µmol, urine IL-8/creatinine concentration to be 88% sensitivity and 69% specificity for the diagnosis of VUR [9]. Nevertheless, some conditions like urinary tract infection (UTI), vaginitis and urinary tract manipulation may result in increased level of IL-8. Since the study is limited to infants, there is a need to extend it to older children.

### MOLECULAR IMAGING with radiolabelled IL-8

The safety of ^99m^Tc-labeled IL-8 in humans and scintigraphy of ^99m^Tc-labeled IL-8 in various experimental models of diseases has been studied^12^. Injection of ^99m^Tc-IL-8 is well tolerated and ^99m^Tc-labeled IL-8 scintigraphy is a promising technique for diagnosis of many clinical conditions [12].

### Detection of pulmonary infections

^99m^Tc-labeled IL-8 scintigraphy can be used to detect pulmonary infections in experimental rabbit models of aspergillosis, pneumococcal pneumonia and Escherichia coli induced pneumonia. ^99m^Tc- IL-8 is valuable for early and excellent visualization of the extent and localization of pulmonary infections. ^99m^Tc- IL-8 is quick and easy to prepare and has the advantage of short time span between injection and imaging. ^99m^Tc- IL-8 offers low radiation burden and is associated with clear delineation of infection foci [13].

### Detection of Osteomyelitis

Rapid diagnosis of osteomyelitis is still challenging. Diagnostic changes appear only at advanced disease stages on imaging films. Bone scanning and scintigraphic imaging with ^99m^Tc-methylene diphosphonate (MDP) are sensitive, but have low specificities. This requires an imaging system which can provide high specificity. Stephen et al evaluated the role of ^99m^Tc-labeled IL-8 in disease model of osteomyelitis [14]. Based on their experimental results, ^99m^Tc- IL-8 can be used to evaluate osteomyelitis with good imaging quality and less radiation burden [14].

Other approaches for diagnosis include; anti-granulocyte monoclonal antibody fragment labeled with Technetium-99m which has recently been proposed for diagnosis of osteomyelitis with high sensitivity and specificity [15].

### Detection of Inflammatory bowel disease (IBD)

Radiolabeled leukocytes are routinely used for imaging inflammatory bowel disease (IBD) but there are many disadvantages associated with this procedure e.g., it is difficult to prepare [16]. Thus, there is a need for an agent with good sensitivity that is easier to prepare. Stephen, *et al *studied the potential of IL-8 labeled ^99m^Tc using hydrazinonicotinamide (HYNIC) for imaging of inflammatory bowel disease (IBD) in rabbit model of acute colitis [16]. They concluded that ^99m^Tc-IL-8 is a promising agent which can be prepared easily and provides excellent imaging with high target-to-background ratios [16]. Crohn's disease (CD) associated mononuclear cell infiltrate can be detected by ^123^l-lL2 scintigraphy [17].

### Marker for Chorioamnionitis

Shimoya, *et al *investigated IL-8 levels in cord sera of babies to explore possible association with chorioamnionitis diagnosis [18]. They found that infected babies had higher IL-8 titer as compared to non infected babies at 22-36 gestational weeks. They concluded that cord serum IL-8 titer is a highly sensitive and specific diagnostic marker as compared to other conventional markers available for diagnosis of chorioamnionitis[18].Among other markers; C-reactive protein (CRP) estimation has 80% sensitivity and specificity for early diagnosis of subclinical chorioamnionitis[19].

### Marker for nasocomial bacterial infections

Nasocomial bacterial infections are responsible for high rate of mortality and morbidity in neonatal patients [20]. Routine investigations and tests used for evaluation of infection are non-specific. There is therefore a need to explore new markers which can detect infection at early stages and can thus allow for immediate commencement of antibiotic therapy after diagnosis to reduce high mortality rate.

Axel, *et al *investigated the role of IL-8 as an early marker for diagnosis of Nasocomial bacterial infections [20]. They found that combination of IL-8 and C-reactive protein (CRP) is able to detect culture proven nasocomial bacterial infection with 96% specificity. They proposed that this combination of markers is cost effective and reliable for early diagnosis of nasocomial bacterial infections in neonates [20]. Joern, *et al *compared plasma levels of IL-8 with IL-6 to check their potential as a marker for infection in neonatal intensive care unit (NICU) patients [21]. They concluded that IL-8 is a more useful marker than IL-6 because it requires less sample volume and less time than IL-6 determination [21].

### Marker for non-Hodgkin's lymphoma (NHL)

There is high incidence of non-Hodgkin's lymphoma (NHL) in western world especially in North America and European countries since past many years [22-24]. There are studies which evaluated the role of IL-8 as a diagnostic marker for non-Hodgkin's lymphoma (NHL) [25]. Hye lin, *et al *measured serum and urine IL-8 levels to check the role in diagnosis of NHL [25]. Their studies indicate that urine IL-8/Cr (creatinine) level may be used as a diagnostic marker for NHL, but found no differences in serum concentrations of IL-8 between NHL patients and controls [25].

### Role in cancer therapeutics

IL-8 has angiogenic, mitogenic and motogenic activities [26]. There are studies that propose that IL-8 helps in cancer progression [27]. IL-8 expression control may be a valuable tool in designing new therapeutics for control of cancer growth and metastasis. Therefore, further research is warranted to fully understand the mechanisms of IL-8 expression [26].

## Conclusions

Interleukin 8 (IL-8) is a promising marker for many clinical conditions and currently being applied by various subspecialties of medicine either for the purpose of rapid diagnosis or as a predictor of prognosis. Nevertheless, IL-8 level increased as a result of many inflammatory conditions, so careful interpretation of IL-8 level is required to make correlation with desired clinical condition's diagnosis or prognosis. A panel consisting of more then one biomarker including IL-8 will be promising diagnostic and prognostic approach for many clinical disorders. Nevertheless, large-scale studies are needed to substantiate the accuracy and outcome of IL-8's usefulness and effectiveness as a biomarker.

## Competing interests

The authors declare that they have no competing interests.

## Authors' contributions

All authors participated in the preparation of the manuscript, and read and approved the final manuscript.
